# Conceptualization and implementation of community social prescribing evaluation: a case study of the co-designed *Connect Local* and *Spark* programs

**DOI:** 10.3389/fpubh.2026.1761023

**Published:** 2026-03-06

**Authors:** Rajna Ogrin, Nadia Corsini, Jodie Scott, Amy Jarvis, Elizabeth Robinson, Sarah Bonell, Judy A. Lowthian

**Affiliations:** 1 Bolton Clarke Research Institute, Melbourne, VIC, Australia; 2 School of Clinical Sciences at Monash Health, Monash University, Clayton, VIC, Australia; 3 Rosemary Bryant AO Research Centre, College of Health, Adelaide University, Adelaide, SA, Australia; 4 School of Public Health and Preventive Medicine, Monash University, Melbourne, VIC, Australia

**Keywords:** case studies, community, complex adaptive system, evaluation, social connection

## Abstract

Social prescribing involves trusted individuals in clinical and community settings identifying non-medical, health-related social needs and connecting people to community-based supports through a collaboratively developed social prescription. Social prescribing operates within a dynamic, complex adaptive system, making evaluation challenging. This study presents two Australian case studies of co-designed social prescribing programs—*Connect Local* in Melbourne and *Spark* in Adelaide—to examine how evaluation can be conceptualized and implemented. Rather than focusing on program delivery, these case studies are used to interrogate the processes, methodological challenges, and system conditions that shape how impact is understood. The evaluation challenges for both initiatives included shared complexities: the need to balance meaningful data collection with individual and community preferences; measuring impact to meet the needs of interest holders; and the evolution of the contexts in which the programs are delivered and their influence on what constitutes ‘success.’ Analyzing the two case studies against assumptions of linear, simple systems highlighted that a shift in how evaluation is conceptualized and undertaken is required. Impacts were not static, discrete, measurable outputs, but dynamic processes shaped by relationships, shared meaning-making, and adaptive capacity. Conventional evaluation frameworks centered on linear logic models and fixed indicators do not effectively capture impacts driven by relationships, community capacity, and adaptive change. Therefore, program success must be reframed as an emergent presence in which outcomes unfold through interactions between individuals, organizations, and wider systems. This study argues for a shift from milestone-based models to ongoing stewardship-oriented approaches that prioritize monitoring patterns, relationships, and adaptive responses. Indicators may need to shift from static quantitative measures to relational indicators that reflect relationship alignment, coherence of working practices, and growth within the networks and relationships. The question this research poses is: *How can evaluators identify and track indicators that remain meaningful* when *both the context and intervention are evolving, and thereby the outcomes are also changing?* By examining the evaluation journeys of *Connect Local* and *Spark*, this study demonstrates the need for methodological approaches that align with complexity, center on community voice, and explain the emergent, co-constructed nature of social connection impacts.

## Introduction

There is growing evidence that social connection is the foundation of holistic well-being. The release of the inaugural World Health Organization (WHO) Commission on Social Connection Report in 2025 (1) marks the need for a significant shift in our primary health system focus from physical and mental health alone to include social health, all three of which are necessary for holistic well-being (World Health Organization, 2006). Strong social connections are associated with reduced inflammation and a lower risk of heart disease, stroke, and dementia (1, 2) and act as a protective factor against mental health issues such as depression, self-harm, and suicide (1–3). Conversely, social disconnection is a known risk factor for physical and mental ill health, contributing to increased morbidity and premature death (1).

Social prescribing is one of many interventions that can promote social connection. It enables trusted individuals in clinical and community settings to identify non-medical, health-related social needs and co-produce *“*a social prescription” that links people to community supports (4). The literature base of social prescribing has expanded rapidly (5), with studies reporting improvements across mental well-being, physical health, motivation, and sociability, as well as reduced loneliness and healthcare utilization (6, 7). However, methodological inconsistencies, short follow-up periods, and limited generalizability, particularly given that the majority of studies originate from the UK primary care settings, constrain the strength of conclusions (5). It remains unclear what works, for whom, and under what conditions (8).

Traditional approaches often conceptualize social prescribing as a simple intervention at the individual level that, if successful, produces individual outcomes (9, 10). Increasingly, researchers are recognizing that social prescribing initiatives operate within and act upon communities (11, 12), aligning with international guidance emphasizing the importance of strengthening community and societal conditions to promote social connection (13, 14). This shift introduces greater complexity to both implementation and evaluation.

To strengthen the social prescribing evidence base, evaluation should be appropriate for the diverse social prescribing models (15, 16) and respond to shifts in understanding of multi-level determinants of health (17). Complexity science offers a useful lens, considering that health and social systems are underpinned by dynamic interactions, multi-level relationships, and continuous adaptation (18). Within this perspective, interventions unfold within unpredictable environments shaped by “human action and interaction” (19), p. 665, meaning making and structural conditions, rather than being oriented to a narrow and individual focus (17, 18).

Co-design is the active collaboration between interest-holders to design solutions to a pre-defined problem (20)—in this case, social connection. It is especially valuable in understanding lived experiences to help develop innovative solutions. Co-design is used to develop social connection activities because social connection is shaped by relational, contextual, and community-level factors that cannot be adequately understood or addressed through expert-driven design alone, making collaborative design essential for creating interventions that are acceptable, relevant, and feasible in practice (19, 20). This study presents two case studies, *Connect Local* and *Spark*, both evidence-based, co-designed community-based models underpinned by social prescribing–by–design (Connect Local) and by program evolution (Spark). *Connect Local* is a social prescribing program for older adults with chronic conditions who are experiencing or at risk of loneliness, social isolation, and/or depressive symptoms. It employs trained community connectors who work one-to-one with participants to identify personally meaningful social goals and support them to access existing local activities, programs, and groups. Conversely, *Spark* is a community-led social connection initiative developed with midlife women and implemented through a neighborhood community center. It delivers low-pressure, drop-in group events and interest-based activities designed and hosted by trained volunteers (“Sparkies”), providing social spaces that help women build new connections, revisit interests, and strengthen ties across generations.

For each case, we outline the contextual factors shaping program development and describe the practical experiences and learnings across implementation and program evaluation. Both project teams increasingly recognized that conventional, linear evaluation approaches were unable to capture the dynamic, relational, and context-dependent nature of social connection interventions, leading to a shift towards complexity science-informed evaluation approaches.

The aim of this paper is to examine how the evaluation of social connection initiatives can be conceptualized and conducted within complex adaptive systems, using the evaluation journeys of *Connect Local* and *Spark* to highlight methodological challenges and opportunities. By analyzing these cases, we identify what evaluators may need to consider when designing approaches that are responsive to complexity, centered on community voice, and capable of capturing emergent, relational, and system-level change. This paper concludes with considerations on how evaluations could be reimagined to better promote social connection and optimize well-being within complex community systems.

## Program context and design

*Connect Local* and *Spark* programs aim to improve social connections within the community. However, there were significant differences in the history, target population, community setting, intervention model, drivers, and implementation of the approaches, as described below.

### Connect local background

The *Connect Local* program concept was the culmination of 8 years of applied research investigating how to promote the independence and holistic well-being of older community members. Findings consistently demonstrated that conventional, individually focused, evidence-based interventions targeting physical and cognitive functioning were poorly aligned with older adults’ priorities. Recruitment and engagement were low, and participants expressed a strong preference for social connection over prescriptive health-oriented programs (21–23). Concurrently, research was increasing on the detrimental effects of loneliness and social isolation (24–26) and the benefits of social prescribing on reducing loneliness and improving health resource utilization (27). Policy developments, including the establishment of the UK’s National Academy of Social Prescribing (28) and the accompanying National Health Service (29) guidance, further reinforced the potential of social prescribing as a structured, community-oriented approach to promote social connection.

Research in the local context had also identified numerous existing programs and activities being offered in the community to promote social connection, but the difficulty was navigating and accessing them (21). This pointed to the need for an enabling, relational mechanism rather than creating new programs. As a result, a community-wide model that actively leveraged existing assets was proposed (30). This conceptual shift, from developing discrete interventions to strengthening system capacity for connection, was foundational to the program’s design.

Building on established collaborations with a local tertiary health service and a sector peak body, the research team secured funding from The Ian Potter Foundation to co-design, implement, and evaluate a community-based social prescribing program within the Glen Eira local government area of metropolitan Melbourne. Funding criteria required a robust evaluation framework and a focus on older adults who had one or more chronic conditions and were at risk of or experiencing loneliness, social isolation, and/or depressive symptoms. *Connect Local* was therefore designed as an enabling intervention: trained community connectors supported participants in identifying meaningful social goals and engaging with existing community activities and programs. The intended impacts were to address loneliness, social isolation, and/or depressive symptoms, to improve holistic well-being, and to reduce avoidable health service resource use.

### *Spark* background

*Spark* originated from a scoping activity in which loneliness in midlife women was identified as a priority issue in women’s health (31). The intent was to facilitate meaningful collaboration among researchers, the health/community sector, and the community by embedding relevant evidence into practice within 2 years.

The project was a collaboration between an academic-community center with a wider project advisory team, including community organizations, government, and academic partners. The aim was to co-design and implement an evidence-based solution that would strengthen the capacity of the community center to support new grassroots community connection initiatives beyond its physical location.

*Spark* was the community’s solution to a design challenge: How can we shape community centers to help prevent and address loneliness for midlife women? The design brief was largely open with some parameters. First, it should build capacity in the community center to support community-driven initiatives to reach new audiences. Second, it should build on the existing strengths of the community center and local spaces/places. Third, it should intervene at the community level rather than the individual level, creating opportunities for connection that are engaging and address a gap. Finally, it should bring people together and strengthen social ties among those who are and those who are not feeling lonely. *Spark* was the ‘brand’ developed for a suite of low-commitment connection events and initiatives that were conceived in co-design workshops, refined in working groups, and tested for feasibility over several months.

## The proposed models

### Connect local

#### Design rationale: traditional service and deficit-focused approach

**Figure 1:**
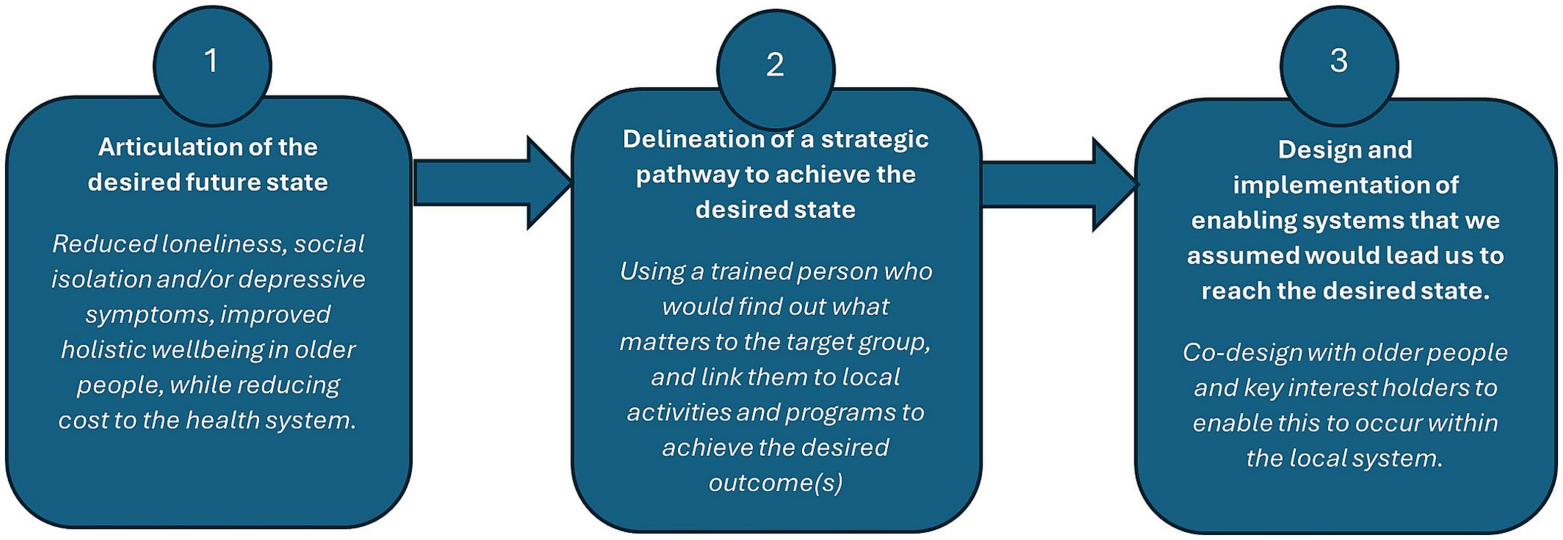
Simple logic approach for *Connect Local* intervention.

At the outset, *Connect Local* adopted a predominantly linear, deficit-focused model due to the external requirements and prevailing service conventions. The funder stipulated that the target population must have at least one chronic health condition and an identified need (loneliness, social isolation, and/or depressive symptoms were proposed). To fulfill the funder’s requirements, the program was initially conceptualized as an individualized intervention providing tailored support to older adults deemed unlikely to initiate social connections independently. This framing aligned with traditional service delivery logics (see Figure 1) but positioned the program within a deficit focus that emphasized individual need rather than community capability.

A structured framework was necessary to guide early design and provide a foundation for evaluation. An existing successful model was selected by the project team, based on general practice in the UK: Health Connections Mendip (27). Although *Connect Local* was to operate within an age and community care context, the Mendip model offered a familiar linear logic: identify need, provide support, achieve individual outcomes; meeting both funder expectations and being aligned with existing organizational goals. The intention was to embed social prescribing within the existing workforce by training nurses to incorporate social needs alongside clinical task delivery, thereby enabling long-term sustainability through integration into routine service delivery.

#### Intervention mechanism

Given that previous research identified numerous existing social connection activities and programs within the community, the research team aimed to engage and refer to existing activities and programs, rather than create new services. This approach was formalized through a simple linear program logic model (see Figure 1), which articulated inputs, activities, and anticipated outcomes in a stepwise sequence consistent with conventional service delivery and evaluation approaches.

#### Implementation context

The implementation context for *Connect Local* was shaped by its delivery within an aged and community care provider, where trained connectors were integrated into existing service structures and expected to support older adults with complex and fluctuating needs. The local community environment included a high density of existing programs but also barriers such as transport limitations, venue accessibility issues, and long waitlists. Local council interest and community enthusiasm facilitated visibility and collaboration, while time pressures to commence implementation before co-design was complete created ongoing adaptation demands. These contextual factors significantly shaped how the program unfolded in practice.

### Spark

#### Design rationale: psychosocial asset-based approach

The team had access to a seed grant that enabled them to scope the project over 6 months. The overarching project goal was informed by insights gathered during an iterative, non-linear scoping process, including interest-holder roundtable discussions, rapid targeted literature reviews, and further interest-holder discussions, to identify project partners and goals (31).

Unlike *Connect Local*, a model could not be identified from the existing evidence. Although epidemiological data supported an upward trajectory of loneliness in midlife (32), no corresponding intervention literature was found. Inspired by the US Surgeon General’s report (33), a public health approach was adopted, focusing on how loneliness can be mitigated among midlife women by leveraging resources within one’s local community or neighborhood. Potential for scale was identified with over 1,000 community centers embedded within communities in Australia (Australian Neighborhood Houses and Centers Association). We reasoned that strengthening the capacity of community centers to engage midlife women could help reduce loneliness in later years.

##### Intervention mechanism

**Figure 2:**
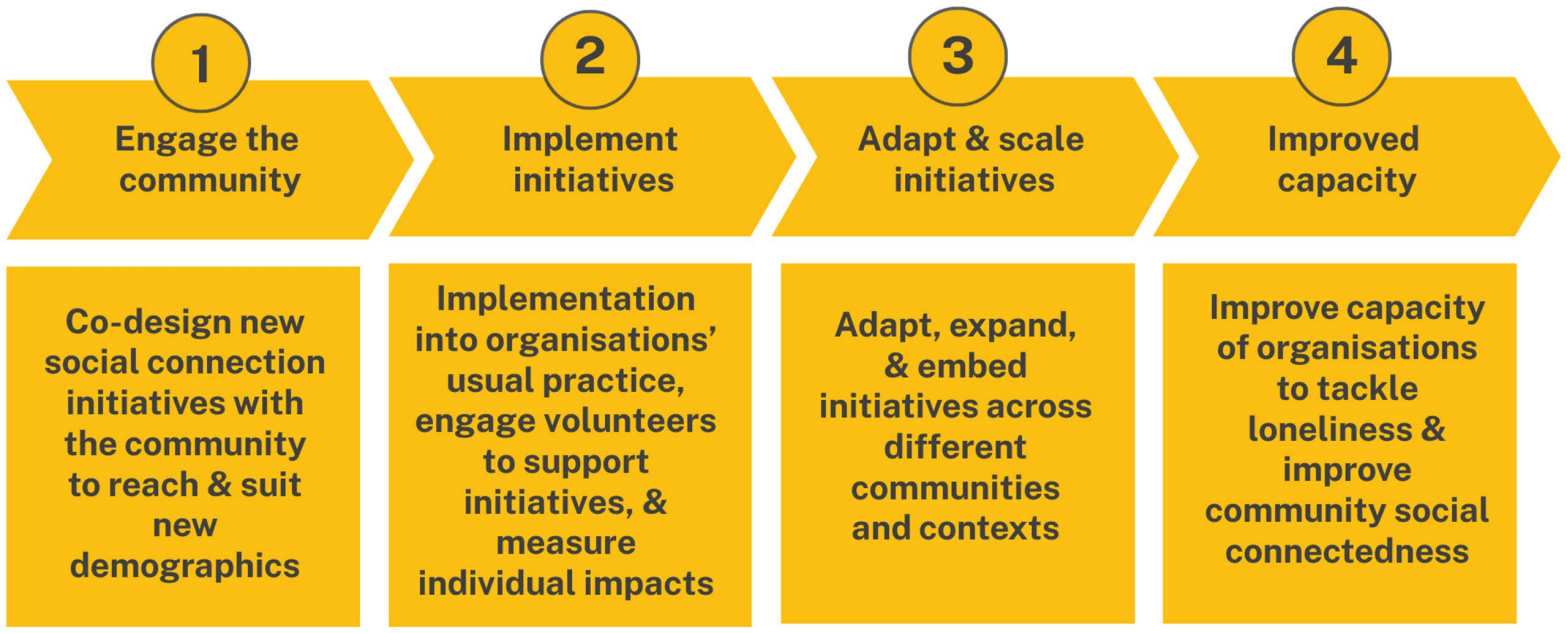
Simple logic approach for *Spark* intervention.

Previous meta-analyses had identified four intervention mechanisms for reducing loneliness: improving social skills, enhancing social support, increasing opportunities for social contact, and addressing maladaptive social cognitions (34). Widely adopted by the WHO (35), these strategies are largely targeted at the individual level and informed our expectations for reducing loneliness across the program. In the absence of targeted intervention evidence, this guided our initial linear approach to the program. The simple linear programming logic used for *Spark* is shown in Figure 2.

##### Implementation context

The funder supported a partnership approach to methodology and evaluation, requiring a demonstration of success in translating knowledge into practice. Ergo, we proposed to use the Knowledge to Action Framework (36) as a process guide and designed the evaluation iteratively as the intervention details were developed. Despite knowing that the intervention would be “unknown,” we hypothesized that it may be educational in nature and that women would value information to raise awareness, reduce stigma, and encourage social connection behaviors. We anticipated that women might be receptive to information presented via an evidence-based podcast series, which the research indicated was a growing communication channel for midlife women. By the end of the co-design workshops, we realized that the preferred model would prioritize action over education. Rather than a static program provider, *Spark* became a community mobilization model, designed to end loneliness by activating volunteers, using existing places and spaces, and building local connection systems. It enables the infrastructure that overlays onto councils, libraries, small businesses, and community groups.

*Note: The authors intentionally use the term *“interest holders”* in place of *“stakeholders”* because the latter carries colonial connotations and is regarded as disrespectful by many Indigenous Peoples (37). Historically, *“stakeholder”* referred to individuals who drove stakes into the ground to mark land they were occupying—often land taken from Indigenous communities (37).

## Program implementation and testing assumptions

### Connect local evaluation

The *Connect Local* funder traditionally prioritized the use of accepted quantitative outcome measures to enable comparability and help address gaps in robust evaluation evidence. In response, the team proposed a rigorous evaluation framework incorporating standardized measures and strengthened this approach to capture impacts at the individual, community, and service levels. To capture contextual factors and implementation processes, qualitative and process evaluation components were also included (shown in Table 1). Theoretical underpinnings were strengthened through the integration of a newly developed social capital theoretical construct (38) and a loneliness model (39), providing a more nuanced basis for understanding change within a community system (40). A detailed description of this evaluation framework is provided elsewhere (40).

**Table 1:** Evaluation of the *Connect Local* program.

Outcome	Level of evaluation	Participants	Measures
Intervention outcome	Individual	Older persons	Loneliness, social isolation, social anxiety, depressive symptoms, well-being and quality of life, goals attained, perceptions of program.
		Service providers	Perceptions of the program
		Volunteers	Well-being, quality of life, and perceptions of the program
	Community	Older persons	Access to activities/services and the number and types of services offered to participants
		Service providers	Impact of the program on community services and satisfaction with the program
	Health System	Older persons	Emergency Department presentations, hospitalizations, hospital length of stay, number of GP consultations, and GP care plan reviews
		Health service providers	Perspectives of staff and satisfaction
Cost effectiveness	Health system	Beneficiaries, Implementers, Promoters, and Funders	Social Return on Investment
Implementation	Intervention, community, and system	Process	Reach, adoption, feasibility, acceptability, appropriateness, fidelity, and sustainability

### *Spark* evaluation

*Spark’s* evaluation model evolved iteratively as the program was implemented, assessing impacts at the individual and community levels, alongside evaluating the co-design process (please see Table 2). Only one of *Spark’s* initiatives (the *Spark* Self Connection 8-week program) was amenable to a traditional pre-post evaluation, which asked participants to report changes in their connection behavior and cognitions. However, it was not appropriate for all other initiatives, which were weekly/fortnightly/monthly events where individuals engaged in a social activity without needing to register. At the community level, the capacity of community centers to support new grassroots connection initiatives was assessed via feedback from community center staff.

**Table 2:** Evaluation of the *Spark* program.

Outcome	Level of evaluation	Participants	Measures
Community engagement process	Individual	Community members Community center staff	Satisfaction with co-design to seed new ideas
Intervention outcomes	Individual	Community members	*Spark Self Connection:* Loneliness; confidence in social connection behaviors and cognitions Impact stories/case studies
		Volunteer Sparkies	Impact stories/case studies
		Community Center staff and volunteers	Confidence to identify and support people who are lonely
Implementation outcomes	Community	NA (process data)	*Acceptability:* Sparkie recruitment, retention and attrition; partnerships with businesses/organizations. *Reach:* Attendance; repeat attendance *Appropriateness:* Professional referrals and outreach. Community referrals and outreach *Adoption:* Implementation of Spark processes into business as usual *Sustainability:* Collaboration requests, grants/funding
	Individual	Volunteer Sparkies	*Sparkie training:* Satisfaction, skills, and confidence to perform the role *Sparkie role:* Satisfaction, enjoyment, challenges, and further training needs

The team adopted a Measurement, Evaluation, and Learning (MEL) approach, in which they sought formal training. This MEL framework, underpinned by a theory of change, has been used across similar place-based evaluations to capture community impacts (41) and centers on cycles of learning and adaptation. The project evaluation used a mixed-methods approach of quantitative measures of initiative engagement and qualitative impact stories. Volunteer community members played a vital role in collecting data at events, using a citizen science approach. Individual impact sessions were also conducted with Sparkie volunteers, but they found it challenging to articulate the impacts they were seeing at events and in the community.

### Evaluation learnings

The approaches utilized by both *Connect Local* and *Spark* were consistent with the assumptions of an intervention delivered within a simple system (42):

1. That the system in question is sufficiently bounded, intelligible, and amenable to control, such that a designed pathway can be reliably executed.
2. That the external environment is sufficiently stable or predictable to permit the articulation of a desirable future state.
3. That it is legitimate to centralize goal and outcome definitions.
4. That the notion of “good” is broadly shared and uncontested across interest holders.

We describe the experiences of *Connect Local* and *Spark* below, related specifically to the above assumptions. It became clear that these assumptions were not met.

*Assumption 1 not met: The system in question is **not** sufficiently bounded, intelligible, and amenable to control such that a designed pathway can be reliably executed.*

At inception, *Connect Local* adopted a linear pathway: identify eligible older adults, assess unmet needs, co-design a social connection plan, facilitate activity participation, and then observe a reduction in loneliness, social isolation, and depressive symptoms. In practice, the system within which *Connect Local* operated was neither sufficiently bounded nor controllable to reliably follow this pathway. The program adapted the eight-component Health Connections Mendip model, a UK social prescribing approach built around community connectors, asset mapping, community development, group-based support, health coaching, and the integration of clinical and non-clinical pathways. To inform this adaptation, the research team undertook literature reviews for each of these eight Mendip components. Yet, due to time constraints, implementation commenced before all of the literature reviews and co-design were completed. As a consequence, several core components, such as referral routes, connector role boundaries, and feedback mechanisms, were iteratively reworked during delivery*, alongside the program delivery and evaluation—*in other words, the program was changing as it was being delivered and evaluated.

Early in the program, community members made it clear they preferred an asset-based approach rather than one emphasizing a deficit focus; for example, the eligibility screening using validated loneliness and social isolation tools was frequently experienced as intrusive, stigmatizing, and unwelcome by both the older people and the connectors delivering the program.

Further, the program’s impact enabled some participants to change their behaviors; however, significant barriers at the individual, community, and societal levels limited participants’ engagement in the program. For example, transport gaps, venue accessibility, changing health status, and caregiving responsibilities all contributed to limiting participant engagement. Moreover, the program’s activities extended beyond participants, influencing the practices of local social care providers and shaping the actions of government representatives at the local and state levels. This was shown by connectors brokering new links between local providers and raising accessibility issues with council officers, producing unanticipated system-level changes not represented in the original logic model. These observations indicate that discrete, end-point measures, underrepresented relational gains (e.g., trust, confidence, navigational knowledge), and system ripples (e.g., new partnerships, practice change).

In comparison, *Spark’s* implementation was entirely influenced by the co-design workshops and follow-up working groups. The women rejected several assumptions that the project team had made. First, they rejected an exclusive focus on ‘midlife’ despite identifying with its challenges. Instead, women responded positively to connecting with women across generations. Second, women deprioritized investment in developing evidence-based information packaged into podcasts. Instead, they wanted opportunities to reconnect with prior interests or develop new ones in group settings, and some wanted to explore connection to self and others more deeply with trained facilitators. Third, women felt that reliance on the community center’s branding may limit the reach of a program to a new audience. Instead, they wanted to create a unique program ‘identity’ that could be co-branded with targeted communication.

Taking a socioecological perspective, Table 3 summarizes influences on the programs at the intrapersonal, interpersonal, community, organizational, and policy levels.

**Table 3:** Socioecological and policy factors affecting *Connect Local* and *Spark* program delivery and outcomes.

Level	*Connect Local*	*Spark*
**Intrapersonal**	Many individuals lacked awareness of the significance of social connection and did not consider their own needs or what was required to enable them to connect socially. Several individuals in the *Connect Local* program experienced personal circumstances that limited their capacity for social connection, including but not limited to deep bereavement, escalating health issues, and cognitive decline.	Sparkies and community members influenced one or more initiatives through their: Willingness to own and drive the initiative, design, and implementation of post-co-design workshops. Interest in volunteering/leading initiatives. Motivation to develop programs based on professional experience. Willingness to initiate further events. Willingness to share their talents/skills to create bespoke events. Preference for drop-in formats over structured programs.
**Interpersonal**	Participants in the *Connect Local* program expressed dissatisfaction with the limited awareness of the initiative by others in the community and agreed to join the Friends of Connect Local campaign to be “sign posters” to promote the program within the community. Local Council actively promoted the program, featuring supportive stories in the community newsletter, facilitating meetings between program team members and meetings with various senior groups and events, and enabling Community Connectors to maintain a monthly presence at local libraries to enhance program awareness.	Received requests for initiatives to suit different community segments. Sparkies became enablers for attendance for some participants. Sparkies offered to support participants outside of Spark. Interactions between Sparkies, participants, and The Hut volunteers strengthened commitment to initiatives. Cross referrals between Spark and other providers, including a community mental health service and a Community Connection service provider.
**Community**	Local social care providers altered their activities to improve social connection, for example: o Having dedicated welcoming people to encourage new attendees to engage. o Holding activities after/before programs to encourage social connection. Local community services began providing social connection programs, including: o A local community health service. o The local council’s healthy ageing team. o Adjacent councils. A tertiary health care collaborator was also considering incorporating social connection within their existing programs.	The Hut Community Center was highly respected and trusted within the local community. Several local businesses and organizations were willing to support the initiatives. Venue characteristics affected initiative success. Local media interest and stories promoted awareness.
**Organizational**	Additional funding opportunities were obtained to continue support of the program. Local, state, and national community organizations collaborated on events, including Loneliness Awareness Week and Neighbor Day. Program leads were invited to present on the program activities by the Municipal Association of Victoria, Ageing Well Network, and others.	The Hut identified funding opportunities to expand initiatives to new demographics and to support pilot testing. The Department of Human Services Community Connections program reached out to discuss collaboration opportunities. Several organizations agreed to partner on a funding application to expand Spark to new regions. Partnership between Spark and a Community Connection provider in an adjacent region.
**Policy: state government level**	Mental health and well-being hubs were developed and implemented by the State Government, considering social prescribing as an early intervention and prevention approach for mental health issues. The social prescribing evaluation project was implemented by the State Government, funding six State program evaluations. Australian Disease Management Association (43), funded by the State Government: o Supported a register that collated community activities across the State. o Promoted education resource sharing. o Developed a social prescribing Community of Practice.	The South Australian Legislative Council carried a motion for an inquiry into loneliness and its impact on South Australians. Ending Loneliness Together’s advocacy work to raise awareness of loneliness and social isolation, including an annual Loneliness Awareness Week. The Department of Human Services developed South Australia’s Plan for Ageing Well.
	NSW, ACT, and QLD: Parliamentary inquiries into loneliness and social isolation.	
**Policy: National peak body activities**	Ending Loneliness Together o Raising awareness of loneliness and social isolation. ASPIRE, Australian Social Prescribing Institute of Research and Education o Consolidating Social Prescribing activities in Australia. Relationships Australia o Freely sharing resources to promote social connection. Royal Australian College of General Practice o Special Interest Group in Social Prescribing. Australian Medical Students Association o Special Interest Group in Social Prescribing. Australian Government Department of Health o Included as action in Australia’s Primary Health Care 10-Year Plan 2022–2032 (44).	
**Societal: international peak body activities**	National Academy of Social Prescribing o Consolidating and sharing the evidence. Global Alliance of Loneliness and Social Isolation o Definitions of loneliness, social isolation, and social connection. World Health Organization o Social prescribing toolkit. o Commission on Social Connection—inaugural report 2025. International Society of Social Capital o A consolidated theory of Social Capital was developed. o The Society became more consolidated, with more webinars, resources, and a conference. Recommendations for health guideline changes to include social connection o To include social connection routinely in cardiovascular risk assessments (45).	
**Societal: research**	Swinburne University-led collaboration o Building social connection resources—increasing the understanding of the complexity of the issue, and the need to reframe to be about social connection rather than deficit and individual-focused loneliness and social isolation, completed in early 2025 (46). Increasing evidence base by researchers globally o Highlighting multiple factors impacting social connection—although the relationship is still not clear.	

Generalizable learnings include the value of iterative, ongoing co-design rather than treating design as fixed and the need to prioritize relational engagement before formal assessment. System-level ripple effects, such as new partnerships and changes in local interest holder engagement, should also be treated as legitimate evaluation outcomes rather than by-products. Overall, evaluation approaches should respond to multilevel barriers and enablers across individual, community, organizational, and policy contexts, rather than relying on rigid, linear models of change.

*Assumption 2 not met: The external environment is **not** sufficiently stable or predictable to permit the articulation of a desirable future state.*

For *Connect Local*, the desired future state of connecting older people to activities in their community depends on highly dynamic factors within the external environment. For more details, please see Table 3, which outlines individual, community, and organizational-level activities.

Participants’ journeys were impacted by events that changed their individual circumstances, including bereavement, health, and cognitive decline, which affected their capacity to engage. In parallel, interpersonal dynamics also shaped implementation. For example, dissatisfaction with low program visibility led participants to co-create a “Friends of Connect Local” campaign to act as community “sign-posters,” complemented by council-driven efforts such as newsletter stories and regular connector presence in libraries, resulting in unplanned adaptations that collectively strengthened the program’s referral base.

Community-level variability led to changes in the availability of social programs, limited transport opportunities due to changes in community transport availability, and some groups modified their practices to welcome newcomers joining activities/programs by appointing dedicated welcome roles and holding activities to enable engaging in connection before and/or after scheduled activities and programs (see Table 3 for more examples). Community health services, council aging teams, and adjacent councils began offering new social connection programs, extending the intervention’s footprint beyond individual prescriptions.

At the organizational-level, new funding opportunities were secured, and multi-organization collaborations (e.g., Loneliness Awareness Week, Neighbor Day) occurred, further shifting roles and workflows. These dynamics indicate that control was distributed and effects “spilled over” from individuals to organizations and local government, contradicting the premise of a linear, unchanging pathway.

Broader policy shifts at state, national, and international levels (as shown in Table 3) continually reshaped the context; therefore, the way the intervention functioned. These unpredictable, multi-level developments made it impossible to define or maintain a fixed future state for the program. In the context of these challenges, it is not possible to achieve an end goal of social connection in all cases.

For *Spark*, the need for an evaluation framework adaptable to complex and dynamic settings became evident as the project evolved from the early concept stage to concept development and early implementation. At the outset, the community was driving what the intervention would look like (within previously outlined parameters). What was originally anticipated to be a pilot intervention with a single community center expanded to include local businesses (cafes), another community center in a different council region, the Community Connections program, mental health services, and community members across multiple council regions. It became clear that the evaluation approach needed to keep pace with the widening boundaries of the *Spark* model. So, a MEL framework emphasizing learning and adaptation cycles was used. The evaluation approach and underpinning theory of change were developed and refined throughout the pilot.

Several elements of system dynamism are generalizable to other social connections or social prescribing interventions operating in complex adaptive systems. Participants’ journeys can vary due to changes in health, bereavement, or caregiving responsibilities, while community-level changes, such as variations in program availability and transport options, create similar unpredictability elsewhere. At the organizational level, new partnerships, cross-sector collaborations, and opportunistic funding are common patterns when interventions gain traction. Broader policy shifts also routinely reshape opportunities and constraints, making fixed end states unrealistic across jurisdictions. Furthermore, it takes time to build these connections to see the impacts. Collectively, these features highlight a generalizable need for adaptive goal-setting, flexible evaluation frameworks, and recognition that interventions will interact with shifting multilevel environments in ways that cannot be fully anticipated in advance, as well as longer timeframes to enable the connections to be built and for the impacts to become evident.

*Assumption 3 not met: That it is **not** legitimate to centralize goal and outcome definitions.*

The *Connect Local* research team realized that the original logic model, which focused on the direct impact of the intervention on selected individual outcomes, was too simplistic. In the early stages of the program, it was anticipated that the direct impact of the intervention (social prescription) would lead to a change in the individual behavior (e.g., engaging in local activities/programs), and this would achieve the desired outcome(s) (e.g., reduced loneliness, social isolation, and depressive symptoms, and improved well-being); a simple linear relationship. Practice data indicated that granular, relational progress was often the most consequential: accepting a welcome call, visiting a venue to assess appeal and fit, or articulating a personally valued interest signaled capability gains not captured by standard scales. Participants reported increased willingness to seek help, greater knowledge of local resources, and a sense of being known within the system, benefits aligned with social capital (38), even when immediate attendance was not feasible. Connectors exercised professional discretion to pace engagement and protect dignity, prioritizing rapport over rigid activity schedules and evaluation targets. These dynamics indicate that discrete, centrally imposed outcome measures may be too blunt to detect otherwise important individual progress. The relational aspects of social prescribing make change much more complex and often slow, with the wider impacts playing an important role. It became evident to the research team that to better reflect the effects of the *Connect Local* program, capturing relational impacts at individual, local, and societal levels would be necessary in complexity-responsive evaluation.

For *Spark,* several emergent factors required the research team to be responsive, which influenced the evaluation approach selected. The co-design workshops identified common barriers to participation among midlife women, including the need to commit in advance and to register or complete paperwork, including surveys. Accordingly, the principle that all *Spark* initiatives are low-pressure and low-commitment was established. This meant that all *Spark* events required attendees to be able to “just turn up,” with confidence that trained Sparkies would warmly welcome them. The adoption of this principle significantly impacted the evaluation approach, as individual-level impacts could not be collected via surveys to avoid conflicting with the established principles. Instead, a citizen-science approach using Sparkie-collected data proved instrumental for monitoring and recording initiative participation and engagement.

As the interventions developed, the research team’s understanding of ‘capacity building’ expanded. Originally, capacity building was conceptualized as relating to community center staff (e.g., upskilling and creating resources for them). However, as volunteer Sparkies developed, it became apparent that this was increasing the community center’s capacity to broaden its reach to new community members and operate beyond its physical space. This further reinforced the importance of using Sparkies as citizen scientists. Additionally, it became apparent that the Sparkies would be key for evaluating ripple effects within the community through qualitative impact stories.

Generalizable to both programs, centrally defined linear outcomes inadequately reflect the full trajectory of change. Instead, relational and capability-building progress, such as increased confidence, willingness to seek help, or exploratory visits to community venues, often provide a more meaningful indication of emerging impact than immediate participation. This underscores the transferable need for evaluation frameworks that incorporate participant-defined goals and fine-grained relational indicators, including trust, navigational knowledge, and perceived belonging. The requirement for staff to exercise discretion in pacing engagement and safeguarding dignity is also likely to apply in other settings, particularly where participants experience vulnerability or a changing health status. More broadly, relational and system-level changes unfold slowly and unevenly, necessitating evaluation approaches that recognize multiple pathways and timescales of change rather than rapid behavioral outcomes.

*Assumption 4 not met: The notion of “good” is **not** broadly shared and uncontested across interest holders.*

The diversity of the interest holders raised multiple possibilities of what is good and what is success, consistent with other research. Older adults typically equate success with achieving personal goals, maintaining independence, and enhancing quality of life (47, 48). Frontline social care providers often prioritize increased participation in community activities and programs, viewing participation as a marker of social inclusion (49). Frontline healthcare providers focus on interventions that demonstrably improve clinical outcomes and the health status of the individuals they serve (50). Whereas decision-makers within healthcare organizations seek alignment with service delivery objectives, ensuring interventions integrate smoothly without disrupting operational priorities (51). Conversely, funders frequently define success in terms of cost-effectiveness and reductions in health system expenditure, emphasizing return on investment (52, 53). These divergent priorities generated design and evaluation trade-offs for *Connect Local* and *Spark*. For example, insistence on pre-event surveys risks deterring those affected by loneliness; relaxing this requirement protected access and equity but reduced standardized data capture. These contrasting priorities underscore the need for frameworks that accommodate multiple conceptions of “value” and “impact,” moving beyond linear outcome measures toward approaches that embrace complexity and negotiated goals (42, 54).

Divergent conceptions of “value” and “success” across interest holders are inevitable rather than exceptional and thereby generalizable. For example, older adults prioritize autonomy and meaningful personal goals; social care providers emphasize participation and inclusion; clinicians focus on clinical change; organizations seek workflow alignment; and funders privilege cost-effectiveness. As a result, the differing priorities generate concrete design and evaluation trade-offs, such as balancing equitable access with the burden of pre-event surveys, and such tensions are likely to arise in any setting where multiple sectors intersect. Consequently, the adoption of evaluation frameworks that accommodate plural and negotiated understandings of impact, rather than relying on singular, centrally defined outcomes, is transferable to other settings.

## Discussion

The Connect Local and Spark case studies illustrate two complementary approaches to strengthening social connections through social prescribing. *Connect Local* emerged within a health and aged care context, shaped by a funding model requiring a clear logic framework, quantitative outcomes, and an identified clinical population. Conversely, *Spark* arose from a community-driven process that prioritized co-design, capacity building, and adaptive learning. While *Connect Local* began with a deficit-based logic and *Spark* with an asset-based approach, both programs evolved through iterative engagement with their communities. Here, we make sense of experiences described in the previous section and recommend solutions to support rigorous, practical evaluation practices.

Both programs confronted the limitations of conventional research evaluation that assumes stable systems, predictable outcomes, and linear pathways to impact. The assumptions underpinning their original designs, that the system is bounded and controllable, that the environment is stable, that goals can be centralized, and that all interest holders have the same notion of good, were each challenged in practice. For *Connect Local*, implementation began before co-design was complete, and evaluation frameworks designed around individual change were not suited to capturing the wider ripple effects on local providers, community organizations, and government interest holders. Meanwhile, *Spark* evolved far beyond its initial design brief as community members, local businesses, and additional community centers joined the initiative. These experiences challenged both program teams to view social prescribing within complex adaptive systems where interventions are emergent, and outcomes are co-produced by multiple interest holders across levels of a system (18, 19). A recommendation is to employ continuous monitoring to track progress, learn, and be flexible to adaptations rather than relying on discrete metrics or predetermined endpoints. For co-designed programs, evaluation approaches and methods should likewise be co-developed with the community members and other interest holders to ensure the evaluation is contextually grounded, meaningful, and fit-for-purpose (15, 20).

Across *Connect Local* and *Spark*, the emergent indicators of program success highlighted the inadequacy of traditional outcome measures for capturing the dynamic, relational, and emergent effects of social connection initiatives. Both teams began experimenting with frameworks such as MEL, which enable reflection and responsiveness, with emerging constructs such as social capital (38) to capture shifts in relationships, trust, and belonging. A challenge common to both teams was the expectations of the wide range of interest holders who held distinct perspectives on what constituted success, from individual well-being and independence to participation/engagement, clinical outcomes, system efficiencies, and community capacity building. A recommendation is to choose evaluation methods that assess impact through patterns of interaction and sense-making rather than through isolated quantitative change.

These findings have implications for how policy leaders, funders, researchers, and organizations conceptualize evidence and impact in social prescribing. Both programs experienced unintended consequences of current research methodology and governance that are grounded in milestone-based reporting and had to pivot to accommodate adaptive and relational changes in their social connection interventions. An alternative approach is to recommend stewardship models that enable ongoing learning and course correction rather than compliance with fixed plans. Funders can play a pivotal role by investing in evaluation capacity-building, supporting flexible funding cycles, and fostering communities of practice that enable shared reflection processes between researchers, practitioners, community members, and community interest holders. Policymakers can also facilitate alignment between local initiatives and national frameworks by recognizing varied legitimate sources of evidence and authority. Such shifts would represent a move from evaluation as accountability to evaluation as collective sense-making—a necessary evolution for working within complex adaptive systems.

These case studies highlight that well-intentioned funder requirements inevitably influence the evaluation methods and indicators selected. These requirements are influenced by a range of factors, including guidelines published by peak bodies. It is common for individual indicators to be recommended even for interventions targeting the community level [e.g., evaluation guidelines for the community (55)]. Whilst necessary for encouraging consistency, collecting individual outcomes may not be feasible or even necessary for interventions that target community assets. We recommend that community-based interventions, evaluation methods, and indicator guidelines promote approaches suited to complex systems. Where discrete indicators are recommended, guidance should also specify the contexts in which such measures are feasible or appropriate to collect.

As community case studies, the insights are presented in the spirit of sharing the associated challenges and learnings. They are relevant beyond the social prescribing literature to public health program implementation and evaluation. Many, if not all, public health programs operate in complex and changeable systems, responding to the constraints, unique resourcing, histories, interest-holders, and goals of the settings. Flexible evaluation approaches enable programs to adapt, evolve, or conclude as appropriate. The challenges encountered by *Connect Local* and *Spark*, as described in this paper, posed real risks to the success of the programs, including undermining trust and participation. It is recommended that public health program evaluation support flexible, innovative approaches to generate meaningful, trustworthy data.

## Conclusion

The experiences of *Connect Local* and *Spark* programs illustrate the benefits of viewing both social prescribing and its evaluation through a complexity lens. The intention is not to claim that adopting a complexity viewpoint will influence the success of a program, but rather to propose that the trajectories of such programs are inherently emergent, negotiated, and relational, unfolding through interactions among individuals, communities, and systems rather than through isolated interventions. Three key take-home messages for practitioners and funders of social prescribing interventions are offered that may help frame evaluation decisions: (1) **Design** interventions using processes that combine theory, literature, and the expertise of diverse interest holders; (2) **Allow** pivots and adaptation, as these strengthen ecological validity without undermining intervention fidelity; and (3) **Map** intervention components to goals and recalibrate them as changes occur to ensure transparency and rigor. These approaches enable program outcomes and the analysis of mechanisms of change to evolve as contexts change.

The central question *(How can we identify indicators of impact in systems that are continually changing as a result of our interventions?)* invites a shift from measuring discrete outcomes to cultivating ongoing learning. Reframing evaluation as stewardship within complex adaptive systems may better support the development of evidence-based public health policies that are both rigorous and responsive to the living systems they aim to serve.

## Data Availability

The datasets presented in this article are not readily available because the research teams are still collecting data for publication in the future. Requests to access the datasets should be directed to rogrin@boltonclarke.com.au for Connect Local and nadia.corsini@adelaide.edu.au for Spark.
